# Prevalence and risk prediction value of tricuspid regurgitation by echocardiography in precapillary pulmonary hypertension

**DOI:** 10.1186/s12890-022-02207-4

**Published:** 2022-11-09

**Authors:** Jiahui Li, Aili Li, Yanan Zhai, Lei Li, Yu Zhang, Aihong Chen, Xincao Tao, Qian Gao, Wanmu Xie, Zhenguo Zhai

**Affiliations:** 1 Department of Cardiology, China-Japan Friendship Hospital, No. 2, East Yinghua Rd, Chaoyang, Beijing, 100029 China; 2 Institute of Respiratory Medicine, Chinese Academy of Medical Sciences, Beijing, China; 3 National Clinical Research Center for Respiratory Diseases, Beijing, China; 4 Department of Pulmonary and Critical Care Medicine, China-Japan Friendship Hospital, Beijing, China

**Keywords:** Precapillary pulmonary hypertension, Tricuspid regurgitation, Prevalance, Risk Prediction

## Abstract

**Background:**

In precapillary pulmonary hypertension (PH), the incidence of different tricuspid regurgitation (TR) degree is poorly defined. The impact of TR severity on pulmonary artery pressure (PAP) assessment and clinical risk stratification in precapillary PH remains unclear.

**Methods:**

A total of 207 patients diagnosed precapillary PH who underwent right heart catheterization (RHC) and echocardiography within 3 days were included. The severity of TR was graded as trace, mild, moderate and severe. Pearson correlation analysis was performed to evaluate the correlation between systolic PAP by echocardiography (sPAP_ECHO_) and mean PAP by RHC (mPAP_RHC_) in different TR degree groups. The impact factors on risk stratification of precapillary PH were analyzed by logistic regression analysis.

**Results:**

The proportion of None, Trace, Mild, Moderate and Severe TR group was 2.4%, 23.7%, 39.1%, 28.5% and 6.3% respectively. Right atrium (RA) area increased gradually with TR aggravation (*p* < 0.001). Moderate and Severe TR group had higher N-terminal pro-B-type natriuretic peptide (*p* < 0.001), right atrial pressure (RAP) (*p* = 0.018), right ventricular basal diameter (RVD)/left ventricular basal diameter (LVD) ratio (*p* < 0.001), larger right ventricle (RV) (*p* < 0.001) and lower tricuspid annular plane systolic excursion (*p* = 0.006) compared with Trace and Mild group. TR-sPAP_ECHO_ in Moderate TR group had the greatest correlation coefficient with mPAP_RHC_ (0.742, *p* < 0.001) followed by Mild (0.635, *p* < 0.001) and severe group (0.592, *p* = 0.033), while there was no correlation in Trace TR group (0.308, *p* = 0.076). Multivariate logistic regression showed three significant independent echocardiography predictors of high-risk precapillary PH: RVD/LVD ratio (OR = 5.734; 95%CI1.502–21.889, *p* = 0.011), RA area (OR 1.054; 95% CI 1.004–1.107, *p* = 0.035) and systolic annular tissue velocity of the lateral tricuspid annulus (S’) (OR 0.735, 95% CI 0.569–0.949, *p* = 0.018).

**Conclusions:**

Precapillary PH was not necessarily accompanied by significant TR. None or Trace TRaccounted for 26% in our population and TR-sPAP_ECHO_ was not applicable to estimate PAP in these patients. RVD/LVD ratio, RA area and S’ can independently predict the high-risk patients with precapillary PH. TR may play an indirect role in risk stratification by affecting these indicators.

## Background

Tricuspid regurgitation (TR) is generally considered highly prevalent and an important prognostic factor in patients with pulmonary hypertension (PH) [1]. Peak velocity of TR (TR Vmax) and its derived parameters systolic and mean pulmonary artery pressure (PAP) by Doppler echocardiography (sPAP_ECHO_ and mPAP_ECHO_) are most commonly used for assessing PH. We previously investigated the accuracy and impact factors of TR in estimating PAP and have demonstrated sPAP_ECHO_ was superior than other TR-related methods in screening PH [2]. However, in precapillary PH, the true incidence of TR and the distribution of different TR degree are still poorly defined. The impact of TR severity on PAP assessment and clinical risk stratification in precapillary PH remains unclear.

This paper we want to investigate: i) the prevalence of TR in precapillary PH; and ii) the impact of TR severity on PAP estimation and clinical risk stratification in precapillary PH.

## Methods

### Subjects

We included consecutive patients admitted to China-Japan Friendship Hospital from November 2015 to October 2020, who were diagnosed precapillary PH by right heart catheterization (RHC) [mean PAP by RHC (mPAP_RHC_) ≥ 25 mmHg, pulmonary artery wedge pressure (PAWP) ≤ 15 mmHg and pulmonary vascular resistance (PVR) > 3WU at rest]. We excluded patients with one of the followings: echocardiography and RHC interval exceeded 3 days; either stenosis of right ventricular outflow tract or pulmonary artery; ventricular septal defect or patent ductus arteriosus; echocardiography images were of poor quality not suitable for analysis; primary valvular disease or pacemaker implantation. Patient’s demographic and clinical data were obtained from electronic medical records of our hospital. The institutional review board of China-Japan Friendship Hospital waived the need for written informed consent from patients as this study was a retrospective analysis of clinically acquired data. The original data will be shared on reasonable request by contacting the corresponding author. This study was approved by the Ethics Committee of China-Japan Friendship Hospital in Peking of China (No.2020–95-K59). Baseline assessment of eligible patients including World Health Organization (WHO) heart function class, the level of N-terminal pro-B-type natriuretic peptide (NT-proBNP) and 6-minute walk distance (6MWD) were recorded.

### RHC

Hemodynamic measurements were performed with a 7F Swan-Ganz catheter Philips Allura X-PERFD20 flat-plate angiography system (Baxter Inc) using Seldinger technique via right internal jugular vein pathway. The system was zeroed and referenced at patients’ heart level as previously described [3]. Right atrial pressure (RAP), systolic PAP (sPAP_RHC_), diastolic PAP and PAWP were measured and cardiac output (CO) was obtained using Fick’s method. mPAP_RHC_ and PVR were calculated using standard formulas.

### Echocardiography

Echocardiographic images were acquired using a GE Vivid E9 or E95 machine (GE Healthcare, General Electric Healthcare) equipped with M5S phased-array transducers. Analysis was performed independently by two blinded investigators using EchoPAC software (GE Healthcare version 201). Two-dimensional and Doppler echocardiography were performed according to the recommendations for cardiac chamber quantification guidelines by the American Society of Echocardiography (ASE) [4]. Specific measurements for right ventricle (RV) related parameters were as follows: area of the right atrium (RA) was measured in an apical four-chamber view; right ventricular basal diameter (RVD) and left ventricular basal diameter (LVD) were measured at end-diastole in the RV-focused apical four-chamber view calculating RVD/LVD ratio; right ventricular free wall thickness (RWT) was measured at end-diastole in the epigastric long-axis image. Echocardiographic assessment of TR involved a systematic and stepwise interrogation of TR from four main views: The RV inflow view from the parasternal long axis, the parasternal short-axis view at the level of the aortic valve, the apical four-chamber view and the parasternal four-chamber view. The color doppler was set to scale 63 cm/s, gain -5 dB and 17 frame per second. Determination of the severity of TR relied on integration of multiple qualitative and semiquantitative measures based on regurgitation jet area and vena contracta width (VC). The TR jet area was assessed from a sector that allowed visualization of the entire RA. The VC was measured at the narrowest diameter of color flow seen immediately beyond the area of flow convergence. The image was optimized by narrowing the sector width to enhance the frame rate and zooming in on the area of interest. The VC was measured as an average during four consecutive cardiac cycles. The severity of TR was graded as trace, mild (jet area < 5 cm^2^, VCTR < 3 mm), moderate (area 5–10 cm^2^, VCTR3-6.9 mm) and severe (jet area > 10cm^2^, VCTR ≥ 7 mm or a ratio of jet area to RA area more than 50%). When the results of jet area and VC were inconsistent, we mainly referred continuity equation method to calculate the TR volume (the blood flow through the tricuspid valve orifice in diastole minus the blood flow through the pulmonary valve orifice in systole). A small amount of TR which could not be detected in every section and regurgitant jet was just limited to the valve annulus with an area less than 2 cm^2^ was defined as Trace TR. TR Vmax was measured and the entire TR spectrum was traced to obtain the peak and mean pressure gradient (TR-PG and TR-mPG). sPAP_ECHO_ and mPAP_ECHO_ were calculated by adding the estimated RAP (eRAP) to TR-PG and TR-mPG respectively. eRAP is divided into three categories (3, 8, and 15 mmHg) based on the inferior vena cava diameter and its respiratory variation [5]. RV systolic function was assessed using multiple parameters, including tricuspid annular plane systolic excursion (TAPSE), systolic annular tissue velocity of the lateral tricuspid annulus (S’) and RV fractional area change (FAC). All parameters were repeatedly measured and averaged. TAPSE/TR-sPAP_ECHO_ ratio was also calculated.

### Risk stratification

We identified high-risk subjects according to a simplified risk stratification in pulmonary artery hypertension (PAH), which was modified based on the 2015 European Society of Cardiology (ESC)/European Respiratory Society (ERS) PH guidelines, published by 2018 World Symposium on PH [6] and also supported by 2021 Chinese guidelines for diagnosis and treatment of PH [7]. This simplified method made the risk stratification more clear, simple and convenient for clinical application. Patients were classified as low (< 5%), medium (5%-10%) or high-risk (> 10%) according to their 1-year expected mortality rate. High risk variables included WHO functional class IV; 6MWD < 165 m; B-type natriuretic peptide (BNP) > 300 ng/L, NT-proBNP > 1400 ng/L or RAP > 14 mmHg; cardiac index (CI) < 2.0L/min/m^2^ or oxygen saturation of mixed venose blood (SvO2) < 60%. Patients with at least 2 high risk variables including CI or SvO_2_ were defined as high risk. We analyzed echocardiographic parameters by logistic regression to investigate the risk prediction value of TR in precapillary PH. We put morphological indexes of right heart (RA area, RV, RVD/LVD ratio, RWT)and left heart [left atrium diameter (LAD), left ventricular internal diameter at end-diastole (LVIDd)], RV function indexes (S’ and FAC), left ventricular ejection fraction (LVEF), TR-sPAP_ECHO_ and TR severity into the equation. Since TAPSE and S’ both reflect the long axis motion of RV free wall and the repeatability of S’ was better than TAPSE in our previous study [8], we just put S’ into the equation.

### Statistical analysis

SPSS 24.0 software was used for statistical analysis (Chicago, IL, USA). The missing data was deleted. Normal distribution was assessed by the Shapiro–Wilk test. Continuous variables were expressed as mean ± standard deviation with normal distribution or as median (interquartile range) for variables without normal distribution. Categorical variables were expressed as frequency and percentage. Comparisons of parameters among four groups were performed by a one-way analysis of variance (ANOVA) when normally distributed or nonparametric test when not normally distributed. Categorical variables were described as frequencies and percentages, which were compared using χ2 test. Probabilities of *P* < 0.05 were considered statistically significant. Pearson correlation analysis was performed to evaluate the correlation between sPAP_ECHO_ and mPAP_RHC_. The impact factors on risk stratification of precapillary PH were analyzed by Multivariate logistic regression analysis. Intraobserver and interobserver reproducibility were assessed in randomly selected 28 subjects. Interobserver reproducibility was tested by two independent observers. Interobserver and intraobserver reproducibility were evaluated by means of intraclass correlation coefficient (ICC).

## Results

### Baseline characteristics

**Fig. 1:**
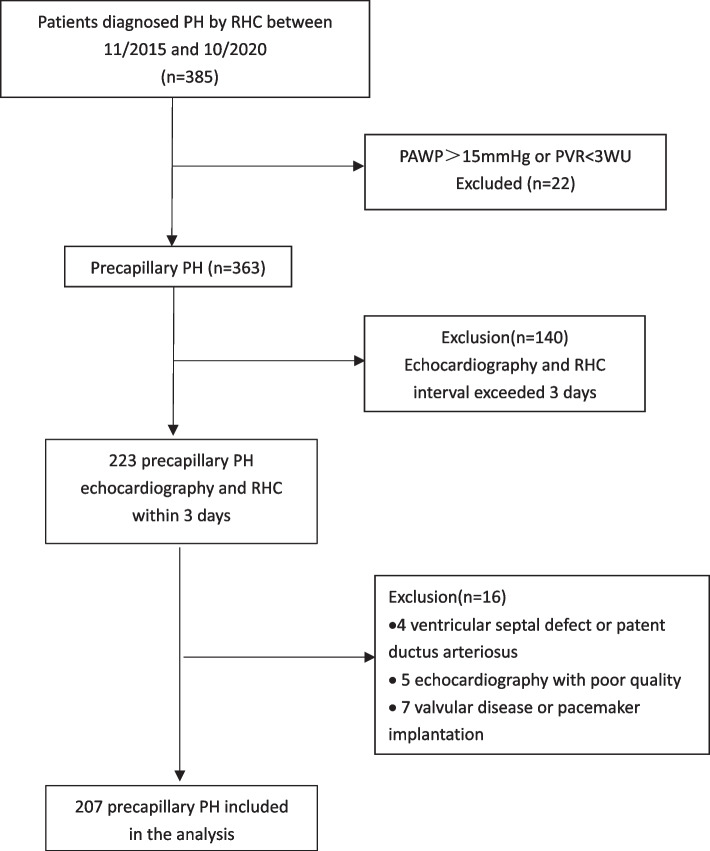
Flow chart of patient screening. RHC: right heart catheterization; PH: pulmonary hypertension; PAWP: pulmonary artery wedge pressure; PVR: pulmonary vascular resistance

**Fig. 2:**
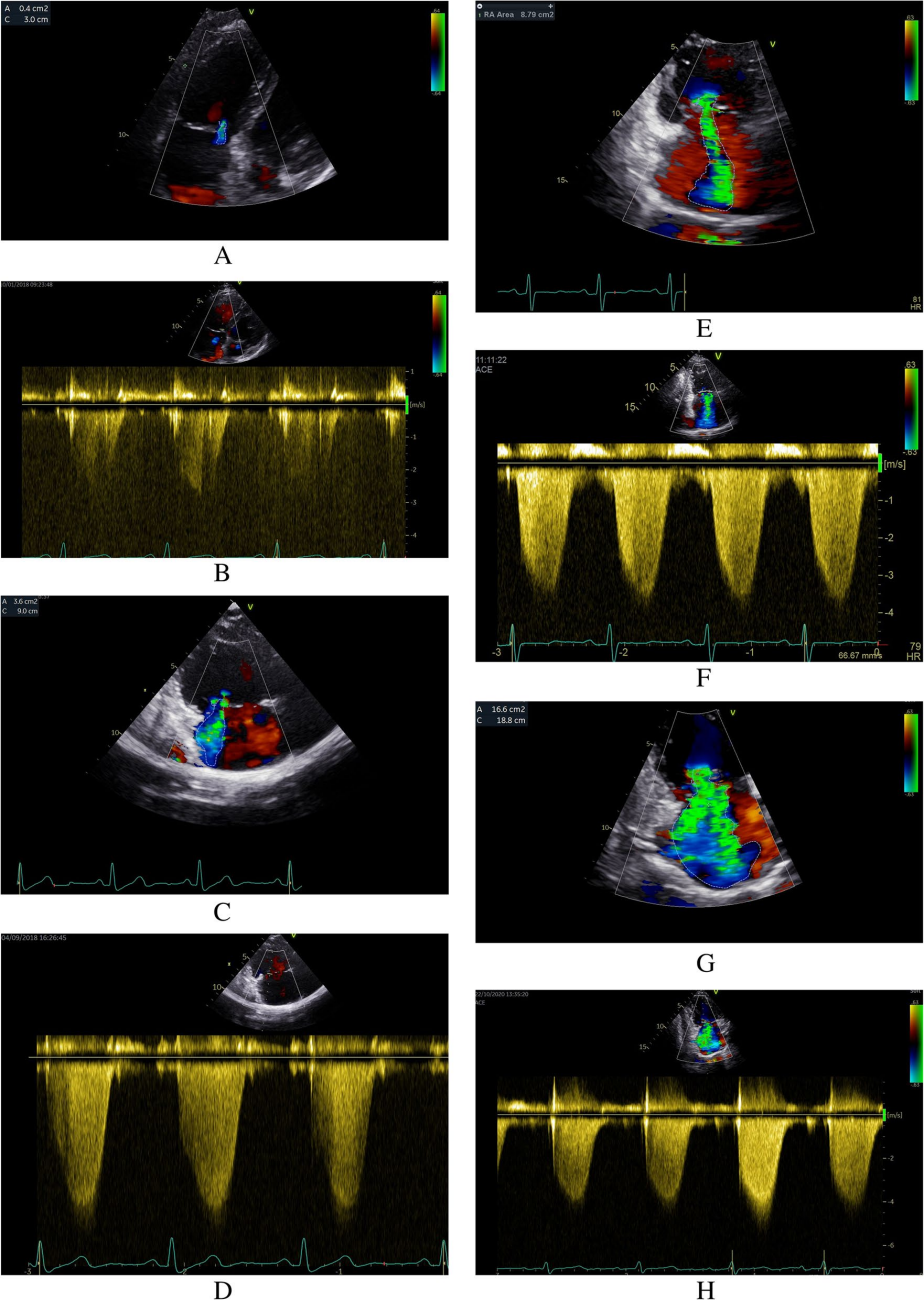
Typical Echocardiography of Color and continuous-wave Doppler in different TR group. **A**, **B**: Trace TR group; **C**, **D**: Mild TR group; **E**, **F**: Moderate TR group; **G**, **H**: Severe TR group. TR: tricuspid regurgitation

**Fig. 3:**
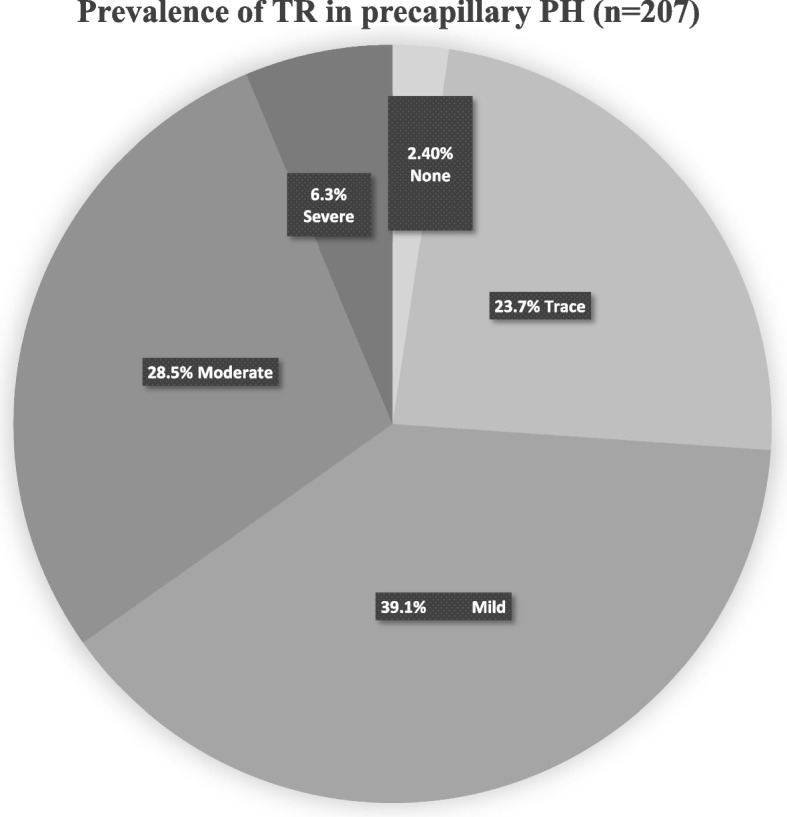
Incidence and distribution of different TR degree in precapillary PH (*n* = 207). TR: tricuspid regurgitation; PH: pulmonary hypertension

**Fig. 4:**
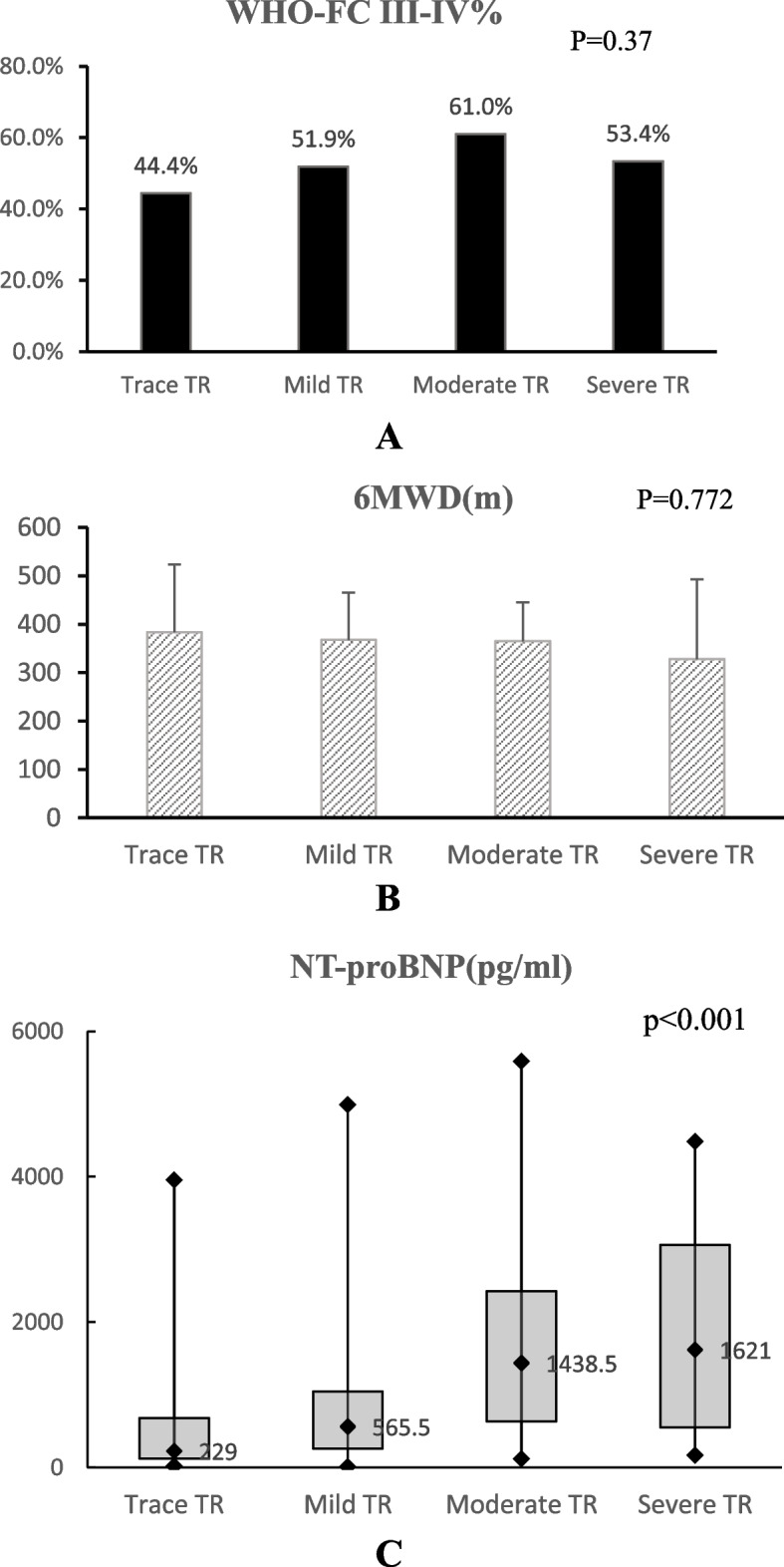
A comparison of WHO heart function III or IV class (**A**), 6MWD (**B**) and NT-proBNP (**C**) among the four groups. TR: tricuspid regurgitation; WHO: World Health Organization; 6MWD: 6-minute walk distance; NT-proBNP: N-terminal pro-B-type natriuretic peptide

A total of 207 patients diagnosed precapillary PH by RHC were finally included as shown in Fig. 1. One hundred one subjects were diagnosed chronic thromboembolic PH (48.8%), 81 were PAH (81, 39.1%), and 25 (12.1%) were lung diseases or of other etiology. Typical images of color and continuous-wave Doppler in different TR groups were shown in Fig. 2. The proportion of None, Trace, Mild, Moderate and Severe TR was 2.4%, 23.7%, 39.1%, 28.5% and 6.3% respectively, as shown in Fig. 3. There were only 5 patients without TR and the number was small. Considering None or Trace TR had similar effects on cardiac structure, function and clinical significance and the difference between them was little, we classified 5 None TR cases into the Trace group. Baseline demographics and clinical characteristics of the four groups were described in Table 1and Fig. 4. There were no differences in age, sex and body surface area (BSA) among the four groups. Although the proportion of patients with WHO heart function III or IV class and 6MWD did not differ among the four groups (*p* = 0.370, 0.772 respectively), NT-proBNP, the sensitive indicator of heart failure increased significantly from Trace TR to Moderate and above (*p* < 0.001). However, there was no significant difference in plasma NT-proBNP level between moderate and severe TR group (*P *= 0.915).

**Table 1:** Clinical characteristics of 4 groups according to TR severity

Baseline characteristics	Trace TR (*n* = 54)	Mild TR (*n* = 81)	Moderate TR (*n* = 59)	Severe TR (*n* = 13)	*P*
Age (years)	48.5 ± 13.9	51 (33,61)	50.3 ± 14.5	45.8 ± 14.7	0.701
Sex (male, %)	44.4%	44.4%	32.2%	46.2%	0.442
BSA (m^2^)	1.7 (1.6–1.9)	1.7 ± 0.2	1.6 (1.5–1.7)	1.7 ± 0.2	0.936
III-IV Class % (WHO Heart function)	24/54, 44.4%	42/81, 51.9%	36/59, 61.0%	7/13, 53.4%	0.370
6MWD (m)	403.4 ± 110.2	368 ± 97.1	365.1 ± 79.9	328 ± 164.6	0.772
NT-proBNP	229.0 (125.0, 681)	565.5* (262.5, 1046.3)	1438.5*♦ (636.5,2426.8)	1621.0*♦ (554.3,3063.3)	*p* < 0.001
PH medical treatment					
Phosphodiesterase type-5 inhibitors	22/54,40.7%	19/81,23.5	13/59,22.0%	4/13,30.8%	0.090
Endothelin receptor antagonists	10/54,18.5%	19/81,23.5%	14/59,23.7%	3/13,23.1%	0.900
Soluble guanylate cyclase stimulator	3/54, 5.6%	5/81, 6.2%	3/59, 5.1%	2/13,15.4%	0.567
Diuretics	44/54, 81.5%	72/81,88.9%	53/59,89.8%	12/13,92.3%	0.477

*TR* Tricuspid regurgitation, *BSA* Body surface area, *WHO* World Health Organization, *6MWD* 6-minute walk distance, *NT-proBNP* N-terminal pro-B-type natriuretic peptide

*p < 0.05 (*p* = 0.006, < 0.001, 0.004 for Mild, Moderate and Severe group respectively) vs Trace TR group; ♦ *p* < 0.05 (*p* < 0.001, 0.017 for Moderate TR and Severe TR respectively) vs Mild TR group

### RHC and echocardiography parameters

RHC and echocardiography parameters were shown in Table 2. There were significant differences among the four groups in sPAP_RHC_, mPAP_RHC_, and PVR (*p* = 0.004, 0.014, < 0.001 respectively), which was mainly for the difference between Trace TR and other TR groups (*p* < 0.05 respectively). Patients with mild to severe TR had higher sPAP and PVR compared with patients with Trace TR. However, it’s not that the more severe the regurgitation, the higher the sPAP and PVR. Moderate and Severe TR group had higher RAP than Trace and Mild TR group (*p *= 0.018). The similar results were also found in TR-sPAP_ECHO_, TR-mPAP_ECHO_ and eRAP (*p* < 0.001, < 0.001, < 0.001 respectively). In the morphology of the right heart by echocardiography, RA area increased gradually with TR aggravation (*p* < 0.001), which was the most obvious. Secondly, Moderate and Severe TR group had larger RV, smaller LVIDd and higher RVD/LVD ratio than Trace and Mild TR group (*p* < 0.001,  < 0.001 respectively). Thirdly, patients with Mild TR and above had a thicker RV wall than those with Trace TR (*p* = 0.006). In RV function by echocardiography, TAPSE in moderate and severe TR group decreased compared with Trace and Mild group (*p* = 0.006). TAPSE/TR-sPAP _ECHO_ ratio representing a non-invasive assessment of RV-PA coupling also decreased with the increase of TR (*p* < 0.001). No statistical differences were found in S’ and FAC.

**Table 2:** RHC and echocardiography parameters of 4 groups according to TR severity

	**Trace TR (54,26.1%)**	**Mild TR (81, 39.1%)**	**Moderate TR (59, 28.5%)**	**Severe TR (13, 6.3%)**	***P***
**RHC parameters**					
sPAP_RHC_(mm Hg)	71.0 ± 22.2	78 (64.5, 92.5)^*^	86.8 ± 24.9^*^	83.8 ± 23.0^*^	0.004
mPAP_RHC_(mm Hg)	37 (30.8, 48.3)	44 (35.5,53)^*^	49.0 ± 15.2^*^	48.8 ± 13.1^*^	0.014
PAWP(mm Hg)	7.5 ± 3.7	6 (4,10)	5.7 ± 4.4	8.8 ± 3.7	0.065
PVR(WU)	8.9 (5.3, 11.9)	10.7 (7.4,14.2)^*^	15.5 ± 7.9^*♦^	13.5 ± 4.5^*^	*p* < 0.001
RAP	2.1 ± 3.6	2 (0,5)	4.2 ± 5.6^*^	6.4 ± 5.5^*♦^	0.018
**Echocardiogarphy**					
RA area (cm2)	18 (16, 19.7)	20.8 (17.6,24.8)^*^	26.1 (21,33)^*♦^	35.9 ± 12.9^*♦•^	*p* < 0.001
RVD (mm)	42 (39, 44.3)	45.2 ± 6.2^*^	48.7 ± 7.2^*♦^	52.5 ± 5.8^*♦^	*p* < 0.001
RVD/LVD	1 (0.95,1.3)	1.2 (1.1,1.4)^*^	1.5 (1.2,1.8)^*♦^	1.7 ± 0.4^*♦^	*p* < 0.001
RWT (mm)	5.0 ± 1.1	5.3 (4.7,6.4)^*^	5.6 (5.1,6.4)^*^	6.2 ± 1.6^*^	0.006
TR-sPAP_ECHO_(mm Hg)	61.4 ± 16.3	78.3 ± 18.8^*^	91.7 ± 19.7^*♦^	88.0 ± 21.9^*^	*p* < 0.001
TR-mPAP_ECHO_(mm Hg)	36.9 ± 8.6	45.9 ± 10.4^*^	55.6 ± 12.8^*♦^	56.8 ± 11.4^*♦^	*p* < 0.001
eRAP(mm Hg)	3 (3,3)	3 (3,3)	8 (3,15)^*♦^	15 (5.5,15)^*♦^	*p* < 0.001
TAPSE(mm)	17.1 ± 3.7	16.3 ± 3.6	15.0 ± 3.4^*♦^	14.4 ± 2.6^*♦^	0.006
S’(cm/s)	9.8 ± 2.8	9.9 ± 2.3	9.1 ± 2.0	8.8 ± 1.8	0.122
FAC(%)	30.9 ± 8.7	29.0 ± 8.4	28.0 ± 6.6	29.8 ± 5.8	0.365
LAD	33.5 ± 5.0	32.8 ± 5.1	32(28,36)	32.8 ± 5.6	0.609
LVIDd	42.3 ± 5.2	40.7 ± 4.8	38.5 ± 6.0^*♦^	36.2 ± 4.0^*♦^	*p* < 0.001
LVEF(%)	71 (66,74)	68.7 ± 5.1	70 (65,72)	70.6 ± 6.7	0.207
Tapse/TR-sPAP_ECHO_	0.31 ± 0.11	0.21 (0.15,0.26)^*^	0.16 (0.13,0.19)^*♦^	0.17 ± 0.05^*♦^	*p* < 0.001

*TR* Tricuspid regurgitation, *RHC* Right heart catheterization, *PAP* Pulmonary artery pressure, *sPAP*_*RHC*_ Systolic PAP by RHC, *mPAP*_*RHC*_ Mean PAP by RHC, *PAWP* Pulmonary artery wedge pressure, *PVR* Pulmonary vascular resistance, *RAP* Right atrial pressure, *RA* Right atrium, *RVD* Right ventricular basal diameter, *LVD* Left ventricular basal diameter, *RWT* Right ventricular free wall thickness, *sPAP*_*ECHO*_ Systolic PAP by echocardiography, *mPAP*_*ECHO*_ Mean PAP by echocardiography, *eRAP* Estimated right atrial pressure, *TAPSE* Tricuspid annular plane systolic excursion, *S’* Systolic annular tissue velocity of the lateral tricuspid annulus, *FAC* Right ventricular fractional area change

*p < 0.05 vs Trace TR; ♦*p* < 0.05 vs Mild TR;• *p* < 0.05 vs Moderate TR

### Reproducibility analysis

The interobserver ICC was 0.884 (95% CI: 0.723–0.953) for VCTR and 0.906 (95% CI: 0.631–0.970) for the TR jet area. The intraobserver ICC was 0.918 (95% CI: 0.832–0.961) for VCTR and 0.921 (95% CI: 0.701–0.975) for the jet area.

### Association between TR-sPAPECHO and mPAPRHC in four groups

In our previous study [2], Bland–Altman analysis demonstrated low bias between RHC and TR-derived parameters with wide limits of agreements. In our patients, TR-sPAP_ECHO_ in Moderate TR group had the greatest correlation coefficient with mPAP_RHC_ (0.742, *p* < 0.001) followed by Mild TR group (0.635, *p* < 0.001) and severe group (0.592, *p* = 0.033). In Trace TR group, 5 had no TR and 15 were with insufficient quality of TR signals, totally accounting for 9.7% of the whole population. We evaluated the correlation between sPAP_ECHO_ and mPAP_RHC_ in other 34 patients and no correlation was seen in Trace TR group (0.308, *p* = 0.076).

### Echocardiography predictors for high-risk precapillary PH

There were 37 high-risk precapillary PH patients. The results of the logistic regression were presented in Table 3. Multivariate logistic regression showed three significant independent echocardiography predictors of high-risk precapillary PH: RVD/LVD (OR = 5.734; 95%CI1.502–21.889, *p* = 0.011), RA area (OR 1.054; 95% CI 1.004–1.107, *p* = 0.035) and S’(OR 0.735, 95% CI 0.569–0.949, *p* = 0.018).

**Table 3:** Echocardiography predictors for high-risk precapillary PH

**Predictor**	**Odds ratio**	**Wald**	**95% CI**	***p*** **value**
RVD/LVD	5.734	6.528	1.502–21.889	0.011
RA area	1.054	4.432	1.004–1.107	0.035
S’	0.735	5.582	0.569–0.949	0.018

*RA* Right atrium, *RVD* Right ventricular basal diameter, *LVD* Left ventricular basal diameter, *S’* Systolic annular tissue velocity of the lateral tricuspid annulus

## Discussion

In our study, we reported the distribution of TR in patients with precapillary PH, and found that up to 26% of patients had only trace or none of TR, even though the PAP and PVR significantly increased and the right heart significantly enlarged. On the one hand, it showed that PH was not necessarily accompanied by significant TR; on the other hand, it also suggested that routine TR-related methods could not be used to estimate PAP in these patients. Meanwhile, we must pay attention to the preliminary screening of PH in combination with other indirect signs and the estimated mean pressure by pulmonary regurgitation (PR), so as to avoid misdiagnosing. In meta-analysis by Wang et al. [9], 33.9% to 56% of the PH patients were without TR. However, in another study by Chen et al. [10], 96.5% of the PH patients had TR. Due to different etiology, classification and severity of PH, the results of TR distribution varied wildly. The patients selected in this study were diagnosed precapillary PH with relatively high PAP and PVR. Mild and moderate TR groups accounted for about two-thirds of our precapillary PH patients, in which TR-sPAP_ECHO_ were highly correlated with mPAP_RHC_ the gold standard for measuring PAP. The correlation coefficient in severe TR group was 0.592 (*p* = 0.033) not as good as Moderate (0.742, *p* < 0.001) and Mild TR group (0.635, *p* < 0.001), while TR-sPAP_ECHO_ could also be used to estimate PAP in severe TR group. We reviewed our 13 patients with severe TR and found that there were no obvious characteristic dagger-shaped spectrum in all of them. The reason may be that TR in our precapillary PH patients was secondary and functional, while dagger-shaped spectrum with an early peak pressure and rapid decay was often seen in intrinsic tricuspid valve disease as shown by Fox et al. [11]. We are not quite sure about this and will further study this phenomenon. There was no correlation in Trace TR group accounting for 26% of our population, therefore TR-sPAP_ECHO_ was not applicable to assess PAP in these patients. Patients with PH are often accompanied by pulmonary artery dilatation and PR. PAP assessment by PR is also a good selection in PH patients, which has been confirmed by our previous findings [12].

We also found that there were no significant differences in WHO heart function class and exercise tolerance (6MWD) among patients with different degree of TR, while NT-proBNP was significantly different among the four groups. It indicated that heart function class and exercise tolerance may depend more on the compensation ability of RV function relative to the increased load. NT-proBNP was significantly related to the degree of TR. TR significantly increased RAP leading to intracardiac volume expansion and filling pressure overload of the right heart. The resulting end-diastolic wall stress prompted ventricular and atrial myocardium to produce more NT-proBNP [13], which is much more sensitive and commonly elevated before patients had exercise intolerance and dyspnea that are neither specific nor sensitive for predicting heart function. In terms of right heart function, the routine parameters S' and FAC had no significant differences in different degrees of TR groups, while TAPSE was lower in patients with moderate and severe TR. On the one hand, it may be because these parameters have a certain load dependency [14] and TR can induce overestimations of RV systolic function to the increased blood volume [15]. On the other hand, it may also suggested that the factors affecting the compensation and decompensation of RV function in PH patients were complex, and TR was not a decisive factor. Preload and afterload, the etiology and length of the disease, and RV-pulmonary artery coupling all affect RV function, which should be comprehensively considered in clinical practice. In our study, TAPSE/TR-sPAP_ECHO_ representing a non-invasive assessment of RV-PA coupling decreased with the increase of TR, which can be obtained from routine indicators, more convenient for clinical use and may be helpful in evaluating RV function.

Previous studies have shown that TR was an important echocardiographic index affecting prognosis [1, 16–18]. Referring to the risk stratification in patients with PAH, we found that the echocardiographic parameters that can independently predict the high-risk patients with precapillary PH were RVD/LVD ratio, RA area and S', which reflected right heart remodeling and RV longitudinal systolic function. The degree of TR was not an independent predictor of high-risk precapillary PH. However, the more severe the TR was, the more obvious the expansion of RA and RV was. RAP and NT-proBNP also increased with TR aggravation. So we believe that TR may play an indirect role in risk stratification of precapillary PH by affecting these indicators.

### Study limitations

A significant limitation of this study is the retrospective nature and the small number of patients from a single-center. So studies involving a larger number of patients will be needed to verify our findings. A selection bias was also in our study since TR-sPAPE_CHO_ was the most convenient and commonly used method as the main screening method to decide whether RHC is indicated or not. In this study, only conventional parameters were used to evaluate RV remodeling and RV function by echocardiography. However, in these subjects all the three-dimensional images and two-dimensional dynamic images that could be used for speckle tracking were also collected. In the future, we will further analyze these data to study the impact of TR on RV function. In clinical practice, echocardiography and RHC could not be performed at the same time. The interval selected for this study was within 3 days so as to minimize its impact on the measurements.

## Conclusions

Precapillary PH was not necessarily accompanied by significant TR. None (2.4%) or Trace TR accounted for 26% in our population and TR-sPAP_ECHO_ was not applicable to estimate PAP in these patients. The correlation coefficient between TR-sPAP_ECHO_ and mPAP_RHC_ in severe TR group was not as good as Moderate and Mild TR group, while TR-sPAP_ECHO_ could still be used to estimate PAP in this group. RVD/LVD ratio, RA area and S' can independently predict the high-risk patients with precapillary PH. TR may play an indirect role in risk stratification of precapillary PH by affecting these indicators.

## Data Availability

The original data will be shared on reasonable request by contacting the corresponding author.

## Abbreviations

TR: Tricuspid regurgitation
PH: Pulmonary hypertension
PAP: Pulmonary artery pressure
RHC: Right heart catheterization
sPAP_ECHO_: Systolic pulmonary artery pressure by echocardiography
mPAP_RHC_: Mean pulmonary artery pressure by right heart catheterization
RA: Right atrium
RAP: Right atrial pressure
RVD: Right ventricular basal diameter
LVD: Left ventricular basal diameter
RV: Right ventricle
S’: Systolic annular tissue velocity of the lateral tricuspid annulus
TR Vmax: Peak velocity of tricuspid regurgitation
mPAP_ECHO_: Mean pulmonary artery pressure by echocardiography
PAWP: Pulmonary artery wedge pressure
PVR: Pulmonary vascular resistance
WHO: World Health Organization
NT-proBNP: N-terminal pro-B-type natriuretic peptide
6MWD: 6-Minute walk distance
sPAP_RHC_: Systolic pulmonary artery pressure by right heart catheterization
CO: Cardiac output
ASE: American Society of Echocardiography
RWT: Right ventricular free wall thickness
VC: Vena contracta
TR-PG: Peak pressure gradient of tricuspid regurgitation
TR-mPG: Mean pressure gradient of tricuspid regurgitation
eRAP: Estimated right atrial pressure
TAPSE: Tricuspid annular plane systolic excursion
FAC: Fractional area change of right ventricle
PAH: Pulmonary artery hypertension
BNP: B-type natriuretic peptide
CI: Cardiac index
SvO2: Oxygen saturation of mixed venose blood
LAD: Left atrium diameter
LVIDd: Left ventricular internal diameter at end-diastole
LVEF: Left ventricular ejection fraction
ANOVA: One-way analysis of variance
BSA: Body surface area
ICC: Intraclass correlation coefficient
